# Incidence and Risk of Proteinuria with Aflibercept in Cancer Patients: A Meta-Analysis

**DOI:** 10.1371/journal.pone.0111839

**Published:** 2014-11-03

**Authors:** Ling Peng, Qiong Zhao, Xianghua Ye, Yun Zhou, Danna Hu, Shusen Zheng

**Affiliations:** 1 Department of Thoracic Oncology, The First Affiliated Hospital, School of Medicine, Zhejiang University, Hangzhou, Zhejiang Province, China; 2 Department of Radiation, The First Affiliated Hospital, School of Medicine, Zhejiang University, Hangzhou, Zhejiang Province, China; 3 Zhejiang Food and Drug Administration, Hangzhou, Zhejiang Province, China; 4 Department of Hepatobiliary and Pancreatic Surgery, The First Affiliated Hospital, School of Medicine, Zhejiang University, Hangzhou, Zhejiang Province, China; Center for Molecular Biotechnology, Italy

## Abstract

**Background:**

Aflibercept is a human recombinant fusion protein with antiangiogenic effects that functions as a decoy receptor to bind vascular endothelial growth factor A. Proteinuria is one of its major adverse effects with a substantial variation in the incidence rate, and the overall risk of proteinuria has not been systematically studied. We performed a meta-analysis of published clinical trials to quantify the incidence and relative risk of proteinuria in cancer patients treated with aflibercept.

**Methods:**

The electronic databases were searched, including PubMed, Embase, Cochrane databases, and ASCO (American Society of Clinical Oncology) abstracts. Eligible studies were phase II and III prospective clinical trials of cancer patients treated with aflibercept with toxicity data on proteinuria. Overall incidence rates, relative risk (RR), and 95% confidence intervals (CI) were calculated using fixed or random effects models depending on the heterogeneity of the included studies.

**Results:**

A total of 4,596 patients with a variety of solid tumors from 16 prospective clinical trials were included for the meta-analysis. The overall incidences of all-grade and high-grade proteinuria in cancer patients were 33.9% (95% CI: 27.3–42.1%) and 7.9% (95% CI: 6.1–10.2%). The relative risks of proteinuria of aflibercept compared to control were increased for all-grade (RR = 1.41, 95% CI: 1.13–1.77) and high-grade (RR = 6.18, 95% CI: 3.78–10.12) proteinuria. The risk of developing all-grade and high-grade proteinuria with aflibercept was substantially higher than that of bevacizumab (all-grade: RR 1.85, 95% CI: 1.63–2.11; high-grade: RR 2.37, 95% CI: 1.84–3.05).

**Conclusions:**

Aflibercept is associated with an increased risk of developing proteinuria. Appropriate monitoring and treatment is strongly recommended to prevent potential renal damage. Future studies are still needed to investigate the risk reduction and possible use of aflibercept in cancer patients.

## Introduction

Angiogenesis is the formation of new blood vessels, which is an important process in the growth of malignant tumors. The predominant regulator of tumor angiogenesis is vascular endothelial growth factor (VEGF) [1]. The continuous expression of VEGF by the tumor makes it a rational target for cancer therapy. Direct inhibition of VEGF by anti-VEGF antibody, VEGF Trap, and VEGF tyrosine kinase inhibitors have demonstrated efficacy in treating various solid tumors.

Aflibercept (Ziv-aflibercept), also know as VEGF Trap, is a recombinant fusion protein comprised of the extracellular domain from VEGFR-1 and VEGFR-2 fused with Fc region of human IgG1. It is a circulating antagonist that binds to VEGF-A, VEGF-B and PIGF (Placental Growth Factor), subsequently preventing their interaction with VEGFR-1 and VEGFR-2, which is a more potent VEGF blocker than bevacizumab [2]. It is currently approved as second-line treatment for patients with metastatic colorectal cancer.

Although aflibercept appears to be well tolerated, as with other anti-angiogenic inhibitor, aflibercept may cause some adverse effects. Asymptomatic proteinuria is common in patients treated with anti-VEGF inhibitors. The recognition and management of proteinuria in cancer patients treated with aflibercept is an important issue since proteinuria may be related with renal damage. The risk factors are not well understood. Because of the limited number of patients in each trial, the overall risk of proteinuria with aflibercept is unclear. Thus, we performed a meta-analysis of prospective clinical trials to determine the incidence and relative risk of proteinuria among cancer patients treated with aflibercept.

## Materials and Methods

### Search Strategy and Study Selection

The electronic databases were searched for studies to include in the meta-analysis, including PubMed, Embase, and Cochrane databases. Abstracts presented at the annual meetings of the American Society of Clinical Oncology (ASCO) were also searched manually. The upper date limit of March 2014 was applied, with no lower date limit. Searches include the terms: (“aflibercept”, OR “VEGF-trap”, OR “AVE0005”) And (“cancer”, OR “carcinoma”, OR “sarcoma”), And (“clinical trial”, OR “randomized controlled trial”). The references cited by the included studies were also used to complete the search.

Aflibercept had been approved for the treatment of patients with previously treated colorectal cancer at a recommended dose of 4 mg/kg every 2 weeks (Q2W). Trials using aflibercept at the approved dosage were included. Clinical trials using aflibercept at doses of 6 mg/kg every 3 weeks (Q3W) were also included to assess the possible increased incidence of proteinuria with these treatments.

Eligible criteria for inclusion in this meta-analysis are: (1) prospective phase II and III clinical trails in cancer patients; (2) participants assigned to treatment with single agent aflibercept at 4 mg/kg Q2W or 6 mg/kg Q3W; (3) the language was restricted in English; (4) data available regarding events or incidence of proteinuria, and (5) if multiple publications of the same trial were retrieved, only the most recent publication (and the most informative) was included. Phase I studies were excluded because of the different drug dosage and the relatively small number of patients on these trials. Abstracts of all candidate articles were read by two independent readers (LP and YZ). Articles that could not be categorized based on title and abstract alone were retrieved for full-text review. Disagreements were resolved by consensus between the two readers. To determine the issue of multiple publications from the same data sets, we checked all author names, clinical trial information, and the time period of patient recruitment of the articles.

### Study Selection

Two investigators independently assessed the eligibility of the articles and abstracts identified by the search, and discrepancies were resolved by consensus. Proteinuria was extracted from the safety and toxicity profile in the primary study. These clinical end points were all recorded according to versions 3.0 of the Common Terminology Criteria for Adverse Events (CTCAE) of National Cancer Institute (http://ctep.cancer.gov/reporting/ctc_archive.html). The CTC version 3.0 describes the grading of proteinuria as follows: grade 1, 1+ or 0.15–1.0 g/24 hrs; grade 2, 2+ to 3+ or >1.0–3.5 g/24 hrs; grade 3, 4+ or >3.5 g/24 hrs; and grade 4, nephrotic syndrome. We included all incidences of proteinuria of grade 1 or above in our analysis.

### Assessment of Risk of Bias

Two authors (LP and YZ) independently assessed the risk of bias in the 5 included randomized controlled trials using RevMan 5.3. Agreements were reached by discussion between the two review authors if there were disagreements on specific items in the studies.

### Data Analysis

Information was retrieved from the primary studies, using a standardized data collection form, including the following items: year of publication, first author, underlying malignancies, number of patients, treatment arm. If data from any of the above categories were not reported in the study, items were treated as “NR (not reported)”. The data of the number of patients with all-grade and high-grade (grade 3 and grade 4) of proteinuria and the number of patients receiving single agent aflibercept were extracted from the toxicity profile. For each study, we derived the proportion and 95% confidence interval (CI) of patients with proteinuria. For studies with a control arm in the same trial, we also calculated and compared the relative risk (RR) of proteinuria. For one study that reported zero events in the control arm, we applied the classic half-integer correction to calculate the RR and variance [3]. Authors of the primary studies were not contacted for additional or unreported information. Between-study heterogeneity was estimated using the χ^2^-based *Q* statistic [4]. Heterogeneity was considered statistically significant when *P*<0.05 or *I* ^2^>50%. If heterogeneity existed, data were analyzed using a random effects model. In the absence of heterogeneity, a fixed effects model was used. To calculate the pooled incidence, an inverse variance statistical method was used. A statistical test with a *P* value less than 0.05 was considered significant. To assess the stability of results, sensitivity analysis was carried out by sequential omission of individual studies. To test for variation in incidence estimates by other factors, we conducted a meta-regression analysis. The presence of publication bias was evaluated by using the Begg's and Egger's tests [5], [6]. All of the calculations were performed by STATA version 11.0 (Stata Corporation, College Station, TX) and Review Manager 5.3 (RevMan version 5.3; Copenhagen: The Nordic Cochrane Centre, The Cochrane Collaboration).

## Results

### Study Selection and Characteristics

**Figure 1:**
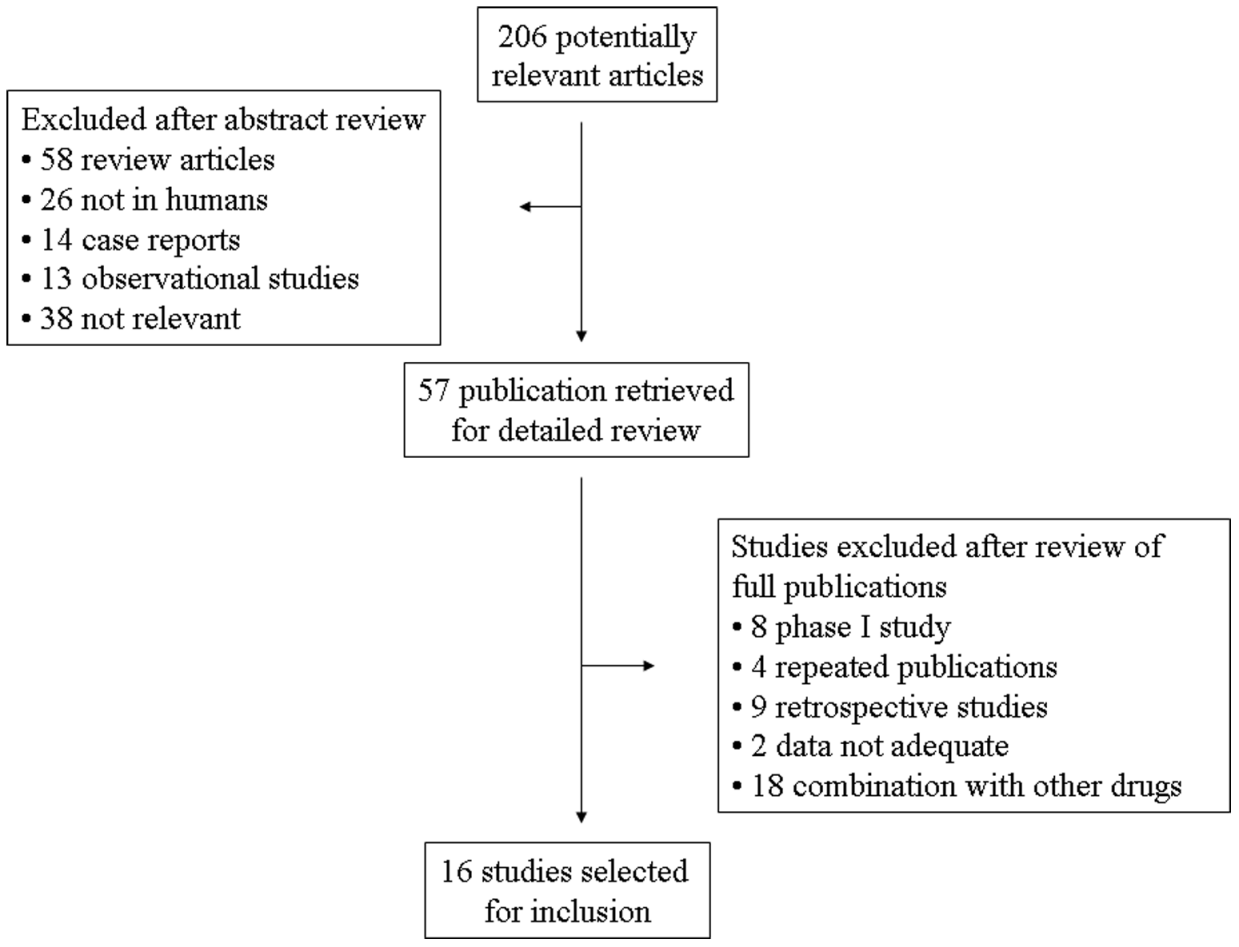
Selection process for the trials included in the meta-analysis.

**Figure 2:**
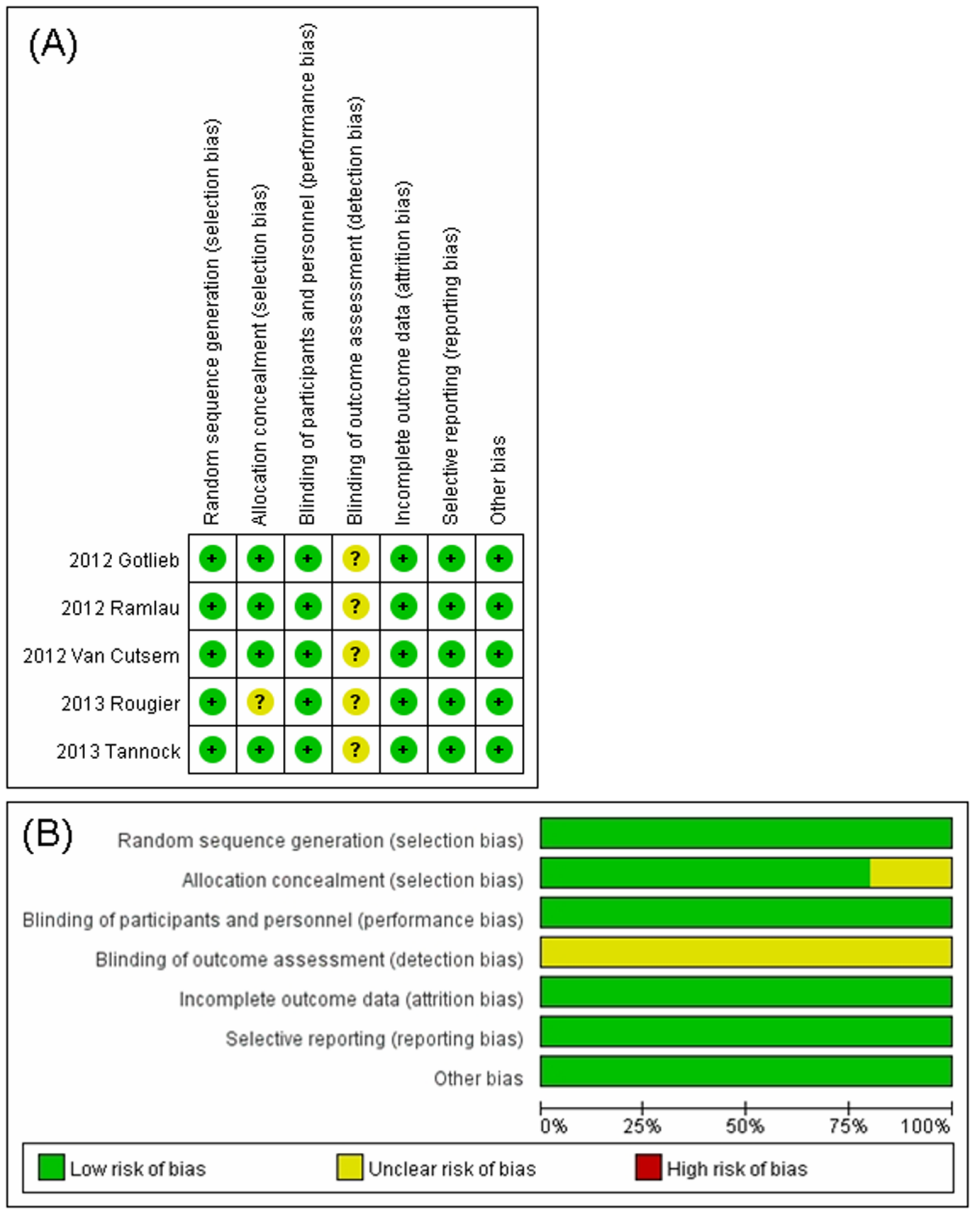
Risk of bias summary and graph. (A) Risk of bias summary. (B) Risk of bias graph. Review of authors' judgments on the risk of bias in each item presented as percentages in the primary studies.

Our search yielded a total of 256 articles on aflibercept from the literature. After reviewing each publication, 15 original studies of full publication met our inclusion criteria. From the abstracts published in American Society of Clinical Oncology (ASCO) meetings, 1 abstracts related to aflibercept were also identified. Altogether, 16 primary studies met inclusion criteria in the search strategy and study selection section, comprising 4596 patients for final analysis (Figure 1). The major baseline characteristics of the 16 eligible studies were reported in Table 1, encompassing 5 randomized controlled trials (RCTs) and 11 phase II clinical trials. Underlying malignancies including ovarian cancer (3 trials) [7]–[9], mCRC (2 trials) [10], [11], non-small cell lung cancer (2 trials) [12], [13], prostate cancer (one trial) [14], pancreatic cancer (one trial) [15], breast cancer (one trial) [16], sarcoma (one trial) [17], endometrial cancer (one trial) [18], melanoma (one trial) [19], glioma (one trial) [20], thyroid carcinoma (one trial) [21], and urothelial cancer (one trial) [22]. The sample size of the included studies ranged from 21 to 611 patients (median sample size, 85 patients). The studies were published between 2010 and 2014. For calculation of the RRs, 5 RCTs were pooled. The risk of bias of the 5 randomized controlled trials was assessed using RevMan and shown in Figure 2. We performed this meta-analysis in accordance with the guidelines of the Preferred Reporting Items for Systematic review and Meta-Analyses (PRISMA) statement [23].

**Table 1:** Main Characteristics and Results of the Eligible Studies.

Study	Year	Phase	Research	Underlying malignancy	Treatment Arm	All-grade	High-grade	Patients
Tew [7]	2014	2	Parallel arm	Ovarian cancer	Aflibercept 4 mg/kg Q2W	22	8	109
					Aflibercept 2 mg/kg Q2W	19	10	106
Tannock [14]	2013	3	RCT	Prostate cancer	Aflibercept 6 mg/kg Q3W	275	38	611
					Placebo	214	7	598
Rougier [15]	2013	3	RCT	Pancreatic cancer	Aflibercept 4 mg/kg Q2W+Gemcitabine	128	14	270
					Gemcitabine	95	3	271
Van Cutsem [10]	2012	3	RCT	mCRC	Aflibercept 4 mg/kg Q2W+FOLFIRI	380	48	611
					FOLFIRI	246	7	605
Tang [11]	2012	2	Single arm	mCRC	Aflibercept 4 mg/kg Q2W	36	8	74
Sideras [16]	2012	2	Single arm	Breast cancer	Aflibercept 4 mg/kg Q2W	NR	1	21
Ramlau [12]	2012	3	RCT	NSCLC	Aflibercept 6 mg/kg Q3W+Docetaxel	34	10	452
					Docetaxel	4	0	453
Mackay [17]	2012	2	Single arm	Sarcoma	Aflibercept 4 mg/kg Q2W	23	4	62
Gotlieb [8]	2012	2	RCT	Ovarian cancer	Aflibercept 4 mg/kg Q2W	18	2	30
					Placebo	16	0	25
Colombo [9]	2012	2	Single arm	Ovarian cancer	Aflibercept 4 mg/kg Q2W	13	0	16
Coleman [18]	2012	2	Single arm	Endometrial cancer	Aflibercept 4 mg/kg Q2W	3	1	44
Tarhini [19]	2011	2	Single arm	Melanoma	Aflibercept 4 mg/kg Q2W	13	6	41
de Groot [20]	2011	2	Single arm	Glioma	Aflibercept 4 mg/kg Q2W	NR	2	58
Twardowski [22]	2010	2	Single arm	Urothelial cancer	Aflibercept 4 mg/kg Q2W	7	1	22
Sherman [21]	2010	2	Single arm	Thyroid carcinoma	Aflibercept 4 mg/kg Q2W	NR	1	21
Leighl [13]	2010	2	Single arm	NSCLC	Aflibercept 4 mg/kg Q2W	16	10	96

Summary table of studies included in the meta-analysis. Abbreviations: CI, confidence interval; NR, not reported.

### Incidence of All-grade Proteinuria

**Figure 3:**
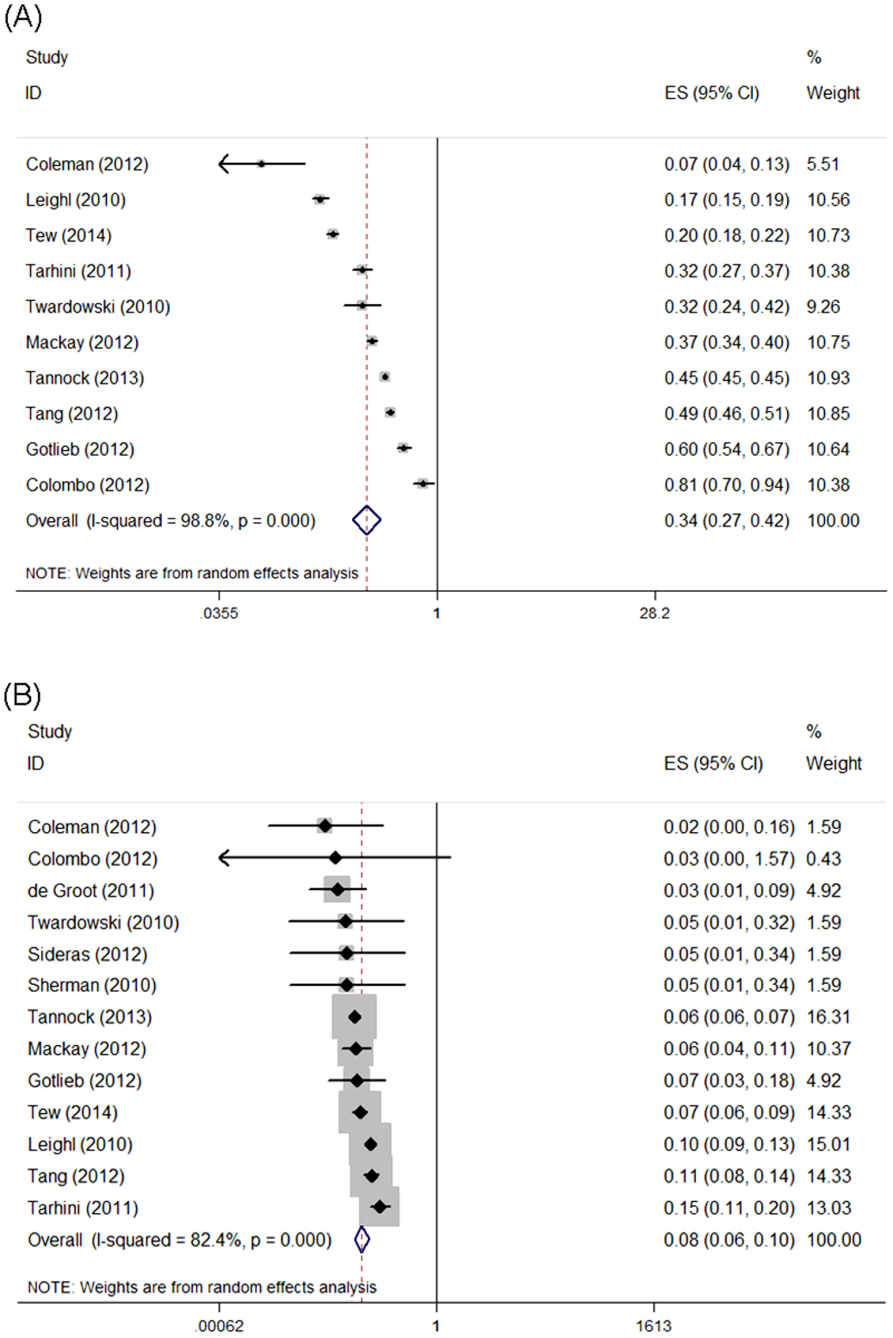
Forest plot for meta-analysis of incidence relative risk of all-grade and high-grade proteinuria in cancer patients treated with aflibercept. Each study was shown by the name of the lead author and year of publication. The summary incidence was also shown in the figure. Plots are arranged as follows: (A) Incidence of all-grade proteinuria; (B) Incidence of high-grade proteinuria.

The results of the meta-analysis were shown in Figure 3. Overall, a total of 4596 patients from 16 trials were included for this analysis. The incidence of all-grade proteinuria ranged from 6.8 to 81%; the lowest incidence was noted in a phase II single-arm trial among patients with endometrial cancer [18], and the highest incidence was observed in patients with ovarian cancer [9]. Our meta-analysis revealed a significant heterogeneity among included studies (*I* ^2^ = 98.8%, *P* = 0.00), and the calculated summary incidence of all-grade proteinuria among patients receiving aflibercept was 33.9% (95% CI: 27.3–42.1%) using a random effects model (Figure 3A). We attempted to quantify the magnitude of potential differences in incidences by those factors by conducting a random-effects meta-regression, and we found that those factors did not seem to affect overall incidence (all *P*>0.2).

### Incidence of High-grade Proteinuria

High-grade proteinuria was associated with significant morbidity, and might result in renal failure and mortality. Thirteen trials reported the incidence of high-grade proteinuria data ranging from 0 to 14.6%. The highest incidence was observed in a phase II trial conducted by Tarhini *et al* in patients with melanoma [19], and the lowest incidence was observed in patients with ovarian cancer [9]. The calculated summary incidence of high-grade proteinuria among patients receiving aflibercept was 7.9% (95% CI: 6.1–10.2%) using a random effects model (*I* ^2^ = 82.4%, *P* = 0.00) (Figure 3B).

### Relative Risk of Proteinuria

**Figure 4:**
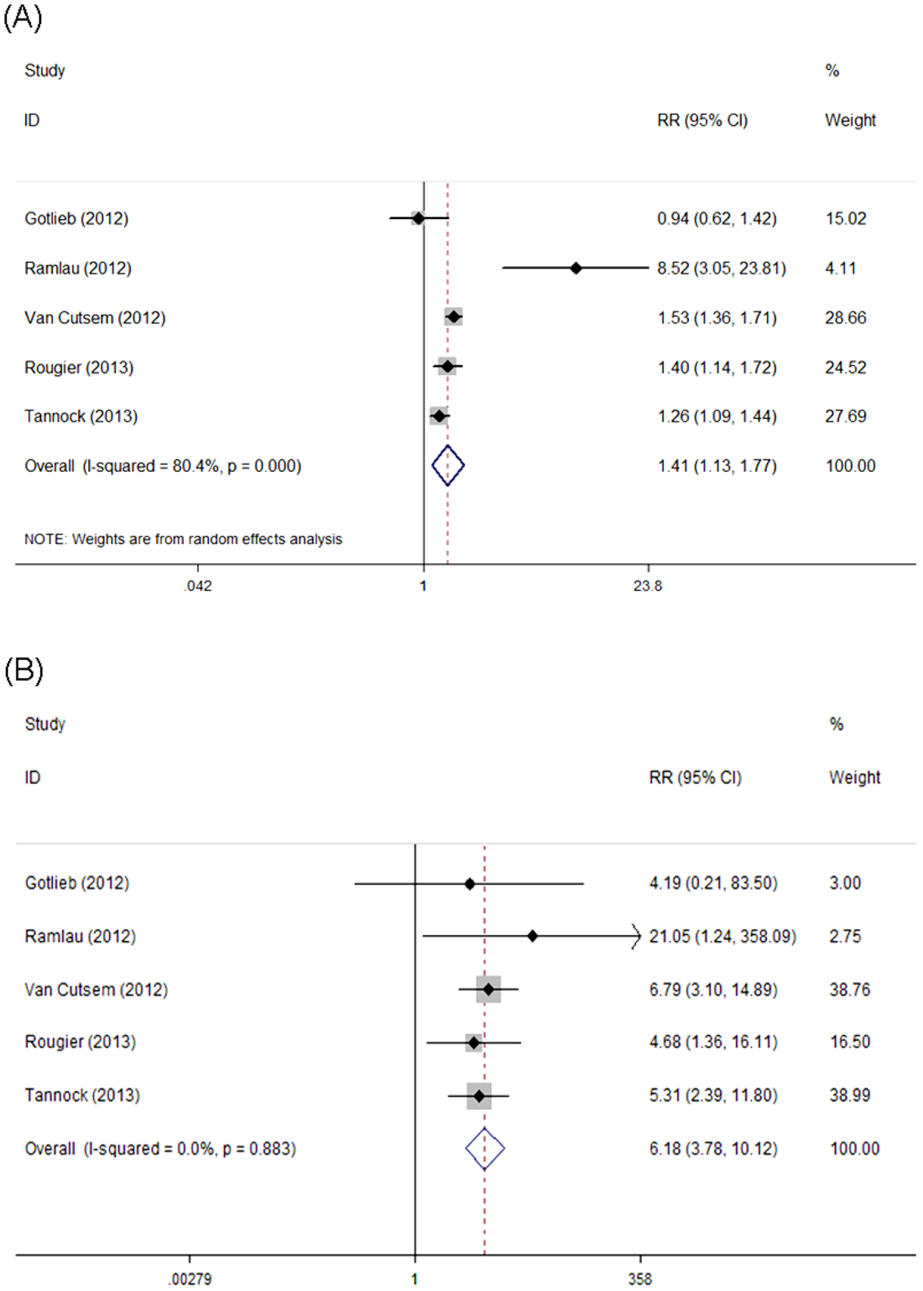
Forest plot for meta-analysis of relative risk of all-grade and high-grade proteinuria in cancer patients treated with aflibercept compared with control. Each study was shown by the name of the lead author and year of publication. Plots are arranged as follows: (A) Relative risk of aflibercept-associated all-grade proteinuria versus control; (B) Relative risk of aflibercept-associated high-grade proteinuria versus control.

With a view to investigate the specific contribution of aflibercept to the development of proteinuria and exclude other therapeutic interventions, we then determined the relative risk (RR) of aflibercept–induced proteinuria compared with control arm. The pooled RR showed that aflibercept treatment increased the risk of developing all-grade proteinuria in cancer patients with a RR of 1.41 (95% CI: 1.13–1.77, *P* = 0.002, Figure 4A) using a random effects model (*I* ^2^ = 80.4%, *P* = 0.00). The incidence for high-grade proteinuria was significantly increased in cancer patients receiving aflibercept compared with control (RR = 6.18, 95% CI: 3.78–10.12, *P* = 0.00, Figure 4B) using a fixed effects model (*I* ^2^ = 0.00%, *P* = 0.88).

We also did sensitivity analysis to examine the stability and reliability of pooled results by sequential omission of individual studies. The results indicated that the significance estimate of pooled incidences and RRs was not significantly influenced by omitting any single study.

### Difference in Proteinuria Incidence Between Bevacizumab and Aflibercept

In addition to aflibercept, other anti-angiogenesis drugs, such as bevacizumab, sorafenib, axitinib, cediranib, and pazopanib have been associated with the development of proteinuria (Table 2). We explored the difference of incidence in proteinuria induced by aflibercept in comparison of bevacizumab. The results showed that the risk of developing all-grade and high-grade proteinuria with aflibercept was substantially higher than that of bevacizumab (all-grade: RR 1.85, 95% CI: 1.63–2.11; high-grade: RR 2.37, 95% CI: 1.84–3.05).

**Table 2:** Incidence and risk of proteinuria with angiogenesis inhibitors.

Drugs	Incidence of proteinuria (95% CI)		Relative risk of proteinuria (95% CI)		References
	All-grade	High-grade	All-grade	High-grade	
Aflibercept	33.9% (27.3–42.1)	7.9% (6.1–10.2)	1.41 (1.13–1.77)	6.18 (3.78–10.12)	Present study
Bevacizumab	13.3% (7.7–22.1)	2.2% (1.2–4.3)	2.79 (1.31–5.95)	4.79 (2.71–8.46)	[32]
Sorafenib	11.6% (4.3–27.6)	0.9% (0.4–1.9)	NR	NR	[33]
Axitinib	20.2% (6.9–46.7)	4.6% (2.2–9.2)	1.24 (0.92–1.68)	5.11 (2.04–12.8)	[33]
Pazopanib	13.5% (3.9–37.6)	2.2% (0.6–6.9)	1.17 (0.88–1.54)	2.69 (1.05–6.91)	[33]
Cediranib	37.8% (27.5–49.3)	3.9% (1.4–10.3)	3.45 (2.41–4.92)	3.63 (1.10–12.03)	[33]

Abbreviations: CI, confidence interval; NR, not reported.

### Publication Bias

**Figure 5:**
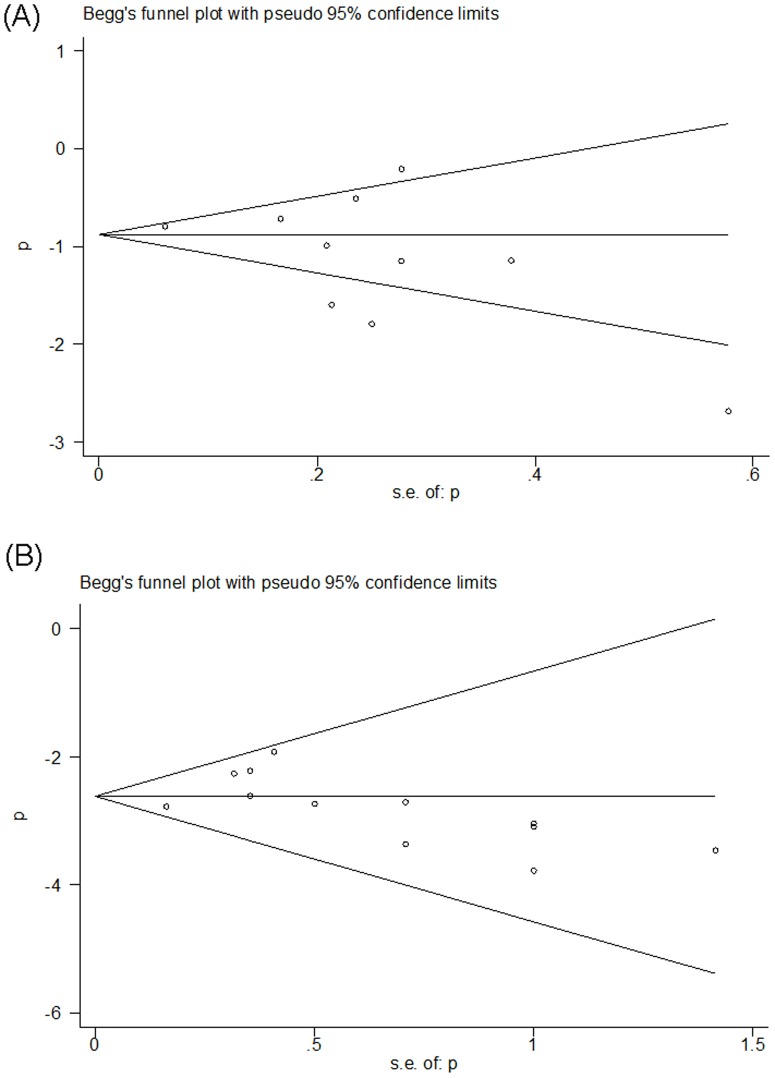
Funnel plot for studies included in the meta-analysis. Plots are arranged as follows: (A) Incidence of all-grade proteinuria in cancer patients treated with aflibercept; (B) Incidence of high-grade proteinuria in cancer patients treated with aflibercept.

Begg's funnel plot and Egger's test were performed to evaluate the publication bias of the eligible studies. Ten and thirteen studies investigating all-grade and high-grade proteinuria induced by aflibercept yielded an Egger's test score of *P* = 0.18 and *P* = 0.45, respectively, indicating the absence of publication bias in the studies (Figure 5).

## Discussion

Angiogenesis, the formation of new blood vessels from existing vessels, is an crucial process in tissue development and growth. Pathologic angiogenesis is a key component of cancer growth and a necessary process for tumor metastasis. Among the proangiogenic factors, VEGF is the most potent and extensively studied. VEGF binding to VEGF receptors (VEGFR1, VEGFR2) initiates angiogenesis signaling process, including increased vascular permeability and endothelial cell proliferation [24]. Antiangiogenic drugs is postulated to block new blood vessel formation and lead to capillary regression [25]. VEGF inhibition is a validated anticancer strategy, and several agents have been designed to target VEGF and angiogenesis pathways.

Aflibercept (VEGF Trap, Ziv-aflibercept, or AVE005) is a recombinant protein consisting of domain 2 from VEGFR-1 fused to domain 3 from VEGFR-2, attached to the hinge region of the Fc domain of IgG1. In contrast to bevacizumab, aflibercept not only targets VEGF-A, but also VEGF-B and PIGF, forming a pharmacologic blockade of the VEGF pathway. Aflibercept has a higher VEGF A binding affinity than bevacizumab [2]. It is approved by the Food and Drug Administration for use in combination with FOLFIRI regimen for second-line treatment of patients with mCRC who have progressed after first-line oxaliplatin-based chemotherapy. Its application in other types of cancer is also undergoing extensive clinical assessment.

Proteinuria is one of the major side effects of this drug, and reported incidences vary substantially among clinical trials. The underlying mechanism is not entirely understood. VEGF plays an important role in regulating glomerular vascular permeability. Treatment of mice with a single dose of anti-VEGF agent resulted in proteinuria [26]. Research suggested that inhibition of VEGF-dependent interactions between podocytes and glomerular endothelial cells disrupts the filtration barrier, which in turn results in dose-dependent proteinuria [27]. Another explanation of proteinuria caused by aflibercept is that inhibition of VEGF signaling pathway induces down-expression of nephrin, sometimes resulting in nephritic syndrome or glomerular thrombotic microangiopathy [28].

The aim of this study is to gain a better understanding of the overall incidence and relative risk of proteinuria in patients with cancer who receive aflibercept. The present meta-analysis has combined 16 publications including 5 randomized controlled trials and 11 phase II trials. Our meta-analysis results demonstrate that aflibercept is associated with an increased risk of developing proteinuria. The overall incidence of all-grade and high-grade proteinuria was 33.9% (95% CI: 27.3–42.1%) and 7.9% (95% CI: 6.1–10.2%), respectively. The relative risks of proteinuria of aflibercept compared to control were increased for all-grade (RR = 1.41, 95% CI: 1.13–1.77) and for high-grade ((RR = 6.18, 95% CI: 3.78–10.12) proteinuria. Data were insufficient to analyze the differences of various underlying malignancies.

We also explore the difference in the incidence of proteinuria associated with aflibercept compared with bevacizumab. The results show that the risk of developing proteinuria with aflibercept is substantially higher than that of bevacizumab. Aflibercept and bevacizumab have different blocking site of the angiogenic pathway. It is possible that the blockade of VEGFR rather than VEGF would result in different downstream effects and toxicities. Since there was no clinical trials which directly compared aflibercept and bevacizumab, the results should be explained with caution. As the development of aflibercept continues, this agent will come to head-to-head comparison with bevacizumab and VEGFR TKIs (sunitinib, sorafenib, pazopanib, cediranib, axitinib, and so on).

Our meta-analysis demonstrates that proteinuria associated with aflibercept is mostly grade 1 and 2. The drug manufacturer recommends monitoring for proteinuria by urine dipstick (or urinanalysis) and determination of the urinary protein-to-creatinine ration (UPCR) prior to each dose of aflibercept [29]. Before administration of aflibercept, patients should be screened for proteinuria. For patients with a UPCR greater than 1, analysis of a 24-hour urine collection is recommended. For patients with high-grade proteinuria, aflibercept should be discontinued and only administered when protein level falls below grade 2 proteinuria, with therapy resumed at a reduced dose of 2 mg/kg Q2W. There is no correlation between the degree of proteinuria and the severity of renal damage, since half of the patients with biopsy finding of thrombotic microangiopathy may have only + to ++ proteinuria on dipstick evaluation [30].

Our meta-analysis has several limitations. One limitation of our meta-analysis is that these studies are conducted at various institutions by different investigators and may have potential bias in reporting the types of adverse events. Secondly, our meta-analysis was based on data from trials that have published results in the literature, but not individual patient data [31]. Thirdly, there was heterogeneity among the primary studies. It is possibly due to different design of the clinical trial and modes of treatment used in each study. In addition, our meta-analysis precludes a more comprehensive analysis such as adjusting for baseline factors and other differences that existed between the trials from which the data were pooled.

In summary, our meta-analysis is the first study to systematically estimate the incidence and relative risk of proteinuria associated with aflibercept in cancer patients. The current analysis suggested that the use of aflibercept increased the risk of all-grade and high-grade proteinuria. The relative risks of proteinuria of aflibercept compared to control were increased for all-grade and high-grade proteinuria. These results would provide important information for clinicians who use aflibercept to treat patients with solid tumors.

## Supporting Information

Checklist S1 **PRISMA checklist.**
(DOC)
